# Increased Risks of Spontaneous Bacterial Peritonitis and Interstitial Lung Disease in Primary Biliary Cirrhosis Patients With Concomitant Sjögren Syndrome

**DOI:** 10.1097/MD.0000000000002537

**Published:** 2016-01-15

**Authors:** Chun-Ting Chen, Yu-Chen Tseng, Chih-Wei Yang, Hsuan-Hwai Lin, Peng-Jen Chen, Tien-Yu Huang, Yu-Lueng Shih, Wei-Kuo Chang, Tsai-Yuan Hsieh, Heng-Cheng Chu

**Affiliations:** From the Division of Gastroenterology, Department of Internal Medicine, Tri-Service General Hospital, National Defense Medical Center, Taipei, Taiwan, Republic of China (C-TC, Y-CT, C-WY, H-HL, P-JC, T-YH, Y-LS, W-KC, T-YH); and Division of Gastroenterology, Department of Internal Medicine, Taipei Medical University Hospital, Taipei, Taiwan, Republic of China (H-CC).

## Abstract

The incidence of Sjögren syndrome (SS) in primary biliary cirrhosis (PBC) patients is high. The influence of SS on the clinical outcomes of PBC patients, however, remains unclear. Our study retrospectively collected data on PBC-only patients and PBC patients with concomitant SS (PBC-SS) to compare the clinical differences of long-term outcomes between them.

A total of 183 patients were diagnosed with PBC from January 1999 to December 2014 at our hospital. Of these, the authors excluded patients with diabetes, hypertension, advanced liver cirrhosis at initial diagnosis of PBC (Child–Turcotte–Pugh classification score of ≥7) and other liver diseases (ie, alcoholic liver disease, alpha-antitrypsin deficiency, viral hepatitis, and primary sclerosing cholangitis), and autoimmune diseases such as systemic lupus erythematosus and rheumatoid arthritis. Of the remaining 125 patients, 77 (61.6%) were PBC-only and 48 (38.4%) were PBC-SS patients.

The mean follow-up duration was 8.76 years. During the observation period, the incidence of interstitial lung disease was higher in the PBC-SS group than in the PBC-only group (*P* = 0.005). The occurrence of spontaneous bacterial peritonitis was significantly different in PBC-SS patients than in PBC-only patients (*P* = 0.002). The overall survival was lower in PBC-SS patients than in PBC-only patients (*P* = 0.033). Although the incidence of hepatocellular carcinoma, end-stage renal disease, variceal bleeding, and hypothyroidism were all higher in the PBC-SS group than in the PBC-only group, the differences were not significant.

Our study suggests that PBC-SS patients have a higher risk of developing interstitial lung disease and spontaneous bacterial peritonitis and have a poor prognosis. Aggressive surveillance of thyroid and pulmonary functions should therefore be performed in these patients.

## INTRODUCTION

Primary biliary cirrhosis (PBC) is a liver disease, with a presumably autoimmune etiology. It is serologically characterized by the presence of antimitochondrial antibodies in 90% to 95% of patients. It often presents with progressive cholestasis of the liver, which results from inflammatory destruction of intrahepatic small bile ducts.^1^ The prevalence and incidence of PBC are increasing, and the female-to-male ratio is approximately 9:1. The most common symptoms are fatigue and pruritus. Ursodeoxycholic acid, the only proven therapeutic agent for PBC, can slow the progression of cirrhosis.^2^ Autoimmune disorders, such as Sjögren syndrome (SS) and systemic lupus erythematosus (SLE), are reported to be associated with PBC. The incidence of SS in PBC patients is reported to range from 21% to 81%.^3^

Sjögren syndrome is a chronic inflammatory autoimmune disease with unknown etiology, often involving the lacrimal and salivary glands. Later in the course of the disease, other organs, such as the lungs, kidneys, liver, cardiovascular system, and central nervous system are also involved.^4^ Dry eyes and dry mouth are the most common manifestations of SS. Recent studies have reported SS patients to be at high risk for the development of autoimmune thyroiditis; the incidence of hypothyroidism is also increased in SS patients.^5,6^ Renal tubular acidosis and renal insufficiency are presentations of renal involvement in SS patients.^7^ The risk of interstitial lung disease (ILD) is also increased in SS patients.^4^

The immune response seen in PBC and SS is a predominance of CD4 + T cell infiltration around the target organ and epithelial transcytosis of IgA against self-antigens.^8^ The mechanisms of autoimmune destruction of PBC and SS are similar, which may indicate frequent coexistence. Both conditions are female predominant, and the mean age at diagnosis is 50 years old. Both PBC and SS can also affect other organs such as the lungs, kidneys, and liver.^4^

The influence of SS on the clinical outcomes of PBC patients remains unclear. In this retrospective study, we aimed to collect the data of PBC-only patients and PBC patients with concomitant SS (PBC-SS patients) to compare the clinical differences of long-term outcomes between them.

## PATIENTS AND METHODS

### Patients

From January 1999 to December 2014, a total of 183 patients meeting the criteria of the American Association for the Study of Liver Diseases Practice Guidelines for PBC at the Tri-Service General Hospital, National Defense Medical Center, Taiwan were diagnosed with PBC.^9^ The diagnosis of PBC included at least 2 of the following criteria: antimitochondrial antibody titer of ≥1:80, abnormal serum alkaline phosphatase (ALP) and gamma-glutamyltransferase (γ-GT) levels for >6 months, and diagnostic liver biopsy.^10^ Patients with diabetes, hypertension, advanced liver cirrhosis at initial diagnosis of PBC (a Child–Turcotte–Pugh classification score of ≥7)^11^ and other liver diseases (ie, alcoholic liver disease, alpha-antitrypsin deficiency, viral hepatitis, and primary sclerosing cholangitis) were excluded. Thus, 136 patients were analyzed in this study. In subgroup analysis, PBC patients were classified into 2 groups: with concomitant SS or without SS. Patients with other autoimmune diseases, such as SLE and rheumatoid arthritis (RA), were excluded. The diagnosis of SS met at least 4 of the following criteria: dry eye for >3 months, dry mouth for >3 months, positive Schirmer test, abnormal salivary gland scintigraphy findings, diagnostic minor salivary gland biopsy, and positive anti-Ro (SS-A) or anti-La (SS-B) antibodies.^12^ At initial diagnosis of PBC, clinical characteristics of patients, including diagnostic age; sex; and levels of serum albumin, serum creatinine, aspartate aminotransferase (AST), alanine aminotransferase, γ-GT, ALP, total bilirubin (TB), thyroid-stimulating hormone (TSH), free thyroxine (free T4), platelet count, international normalized ratio, and AST-to-platelet ratio index (APRI), were analyzed. Our study was reviewed and approved by the Institutional Review Board of the Tri-Service General Hospital (IRB: 1–104–05–065).

### Follow-up and Major Events

All patients were followed up at our hospital and underwent regular laboratory investigations and chest radiography every 3 months. An abdominal ultrasonography was performed every 6 months. If patients presented with progressive dyspnea, an echocardiography and pulmonary function tests (PFTs) were conducted. Ursodeoxycholic acid was prescribed for all patients to prevent the progression of cirrhosis.^2^ Primary biliary cirrhosis-Sjögren syndrome patients were administered oral hydroxychloroquine, as well as artificial tears and pilocarpine for dry eyes and dry mouth. During the observation period, major events, including the incidence of hepatocellular carcinoma (HCC), ILD, end-stage renal disease (ESRD), spontaneous bacterial peritonitis (SBP), variceal bleeding, hypothyroidism, and death, were recorded. Hepatocellular carcinoma was diagnosed according to histopathologic examination of liver biopsy samples or the typical demonstration of HCC based on dynamic abdominal computed tomography or magnetic resonance imaging.^13^ Management of HCC was based on the guidelines for HCC reported by the American Association for the Study of Liver Diseases.^14^ Interstitial lung disease was diagnosed when the following criteria were met: abnormal PFTs, including evidence of restriction, increased alveolar–arterial oxygen tension gradient at rest or during exercise and decreased diffusing capacity of the lungs for carbon monoxide; chest radiography or high-resolution computed tomography showing usual interstitial pneumonitis; and the absence of the following conditions: pulmonary tuberculosis, cardiopulmonary diseases, bronchial asthma, lung cancer, sarcoidosis, bronchiectasis, emphysema, or smoking.^15^ Corticosteroid was administered in patients with ILD, and a PFT was scheduled every 6 months. The definition of ESRD included a level of glomerular filtration rate <15 mL/min/1.73 m^2^ and initiation of renal replacement therapy.^16^ The diagnosis of SBP was made as per the following criteria: ascitic fluid analysis showing polymorphonuclear count >250 cells/mm^3^; a positive ascitic fluid culture; and the absence of other etiologies of secondary peritonitis, including hollow organ perforation, appendicitis, diverticulitis, cholangitis, or cholecystitis.^17^ A 7-day course of antibiotic treatment was administered for SBP control. Variceal bleeding included gastric and esophageal varices hemorrhage, which were confirmed by esophagogastroduodenoscopy and managed using endoscopic variceal ligation (EVL). Beta-blockers were used to prevent rebleeding.^18^ Hypothyroidism was diagnosed when TSH level was above and free T4 level was below the normal range, irrespective of clinical symptoms. Levothyroxine was prescribed for hypothyroidism.^19^

### Statistical Analyses

Continuous variables were expressed as means ± standard deviation, and categorical variables were expressed as percentages. The differences in continuous and categorical variables were analyzed by the Student t test and the χ^2^ test, respectively. The cumulative incidence of disease and overall survival curves were computed using the Kaplan–Meier method and log-rank test. All data analyses were performed using SPSS software version 18.0 (SPSS Inc, Chicago, IL). A *P* < 0.05 was considered statistically significant for all tests.

## RESULTS

### Clinical Characteristics and Long-term Outcomes of All Patients With Primary Biliary Cirrhosis

The clinical characteristics at initial diagnosis of all 136 PBC patients are summarized in Table 1. They included 23 men and 113 women (median age, 47.90 ± 7.42 years). Eight (5.9%) patients had concomitant SLE, 48 (35.3%) had SS, and 3 (2.2%) had RA. The results of laboratory investigations are shown in Table 1. The progression states of liver fibrosis between PBC-only and PBC-SS groups were calculated by APRI values. Aspartate aminotransferase-to-platelet ratio index was a noninvasive biomarker to evaluate liver fibrosis status. Its values <0.5 represented no significant liver fibrosis. Also, its values between 0.5 and 1.5 represented progressive liver fibrosis, and values >1.5 represented advanced liver fibrosis or cirrhosis.^20,21^ At initial diagnosis of PBC, the mean APRI value was 0.71 and 5 (3.7%) patients had APRI values >1.5. The long-term outcomes of PBC patients are shown in Table 2. The mean follow-up duration was 8.76 years. During the observation period, death from all causes occurred in 14 (10.3%) patients. Hepatocellular carcinoma developed in 5 patients; of these, 4 patients were diagnosed as HCC by dynamic abdominal imaging and 1 by liver biopsy. Interstitial lung disease occurred in 15 (11.0%) patients. Eight (5.9%) patients’ renal function progressed into ESRD and renal placement therapy was initiated. The episodes of SBP occurred 18 times in 15 patients, and all patients were managed with antibiotics. The events of variceal bleeding developed 9 times in 8 patients, and EVL was successfully performed in all patients. No patients died because of variceal bleeding. Nine (6.6%) patients developed hypothyroidism, which was controlled using levothyroxine.

**TABLE 1:**
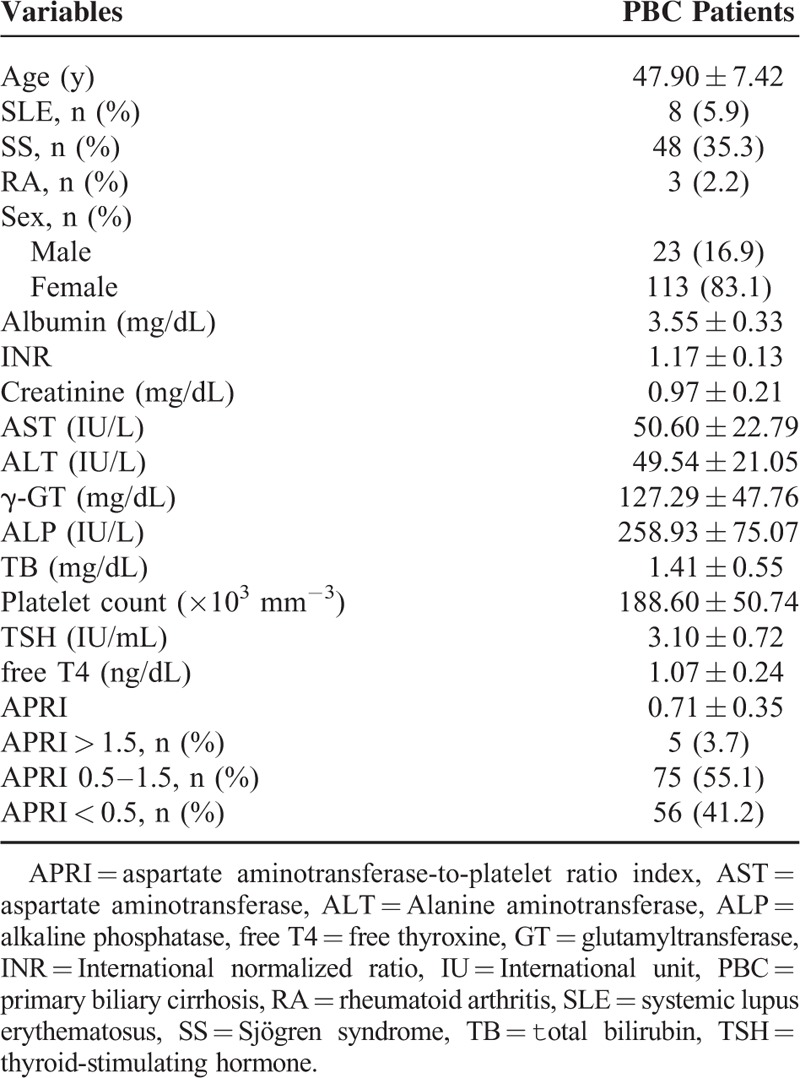
Clinical Characteristics of Primary Biliary Cirrhosis Patients (n = 136)

**TABLE 2:**
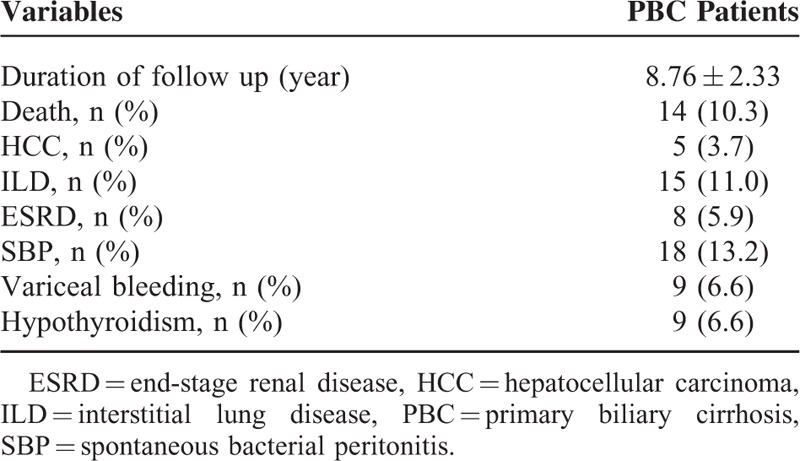
Long-term Outcomes of Primary Biliary Cirrhosis Patients (n = 136)

### Clinical Characteristics and Long-term Outcomes between Primary Biliary Cirrhosis-only Patients and Primary Biliary Cirrhosis-Sjögren Syndrome Patients

**FIGURE 1:**
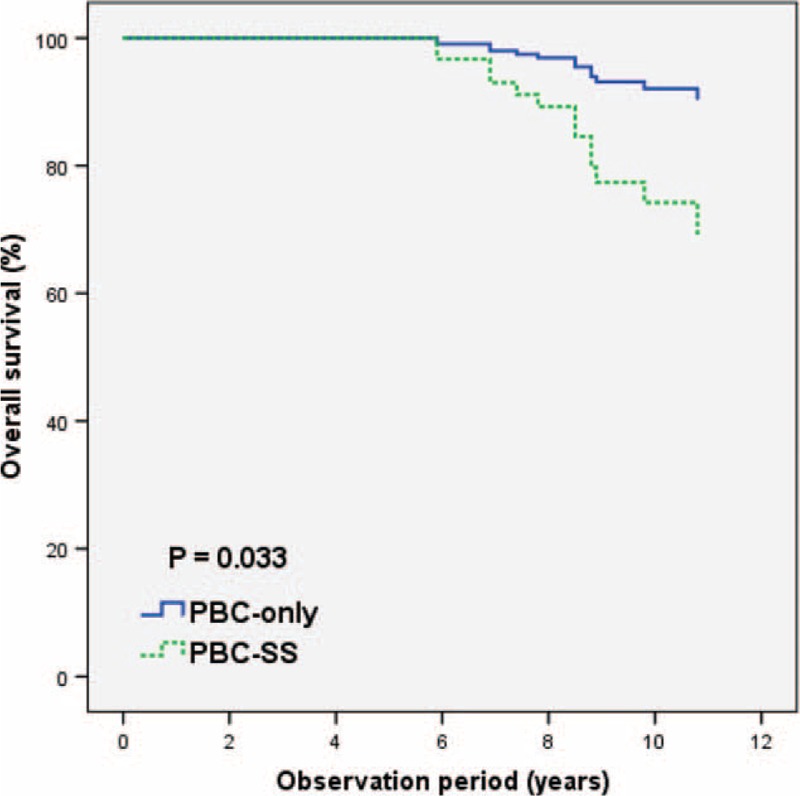
The overall survival curves based on Kaplan–Meier method was significantly lower in primary biliary cirrhosis-Sjögren syndrome patients than in primary biliary cirrhosis-only patients. *P* = 0.033. PBC: primary biliary cirrhosis. PBC-SS: primary biliary cirrhosis with concomitant Sjögren syndrome.

**FIGURE 2:**
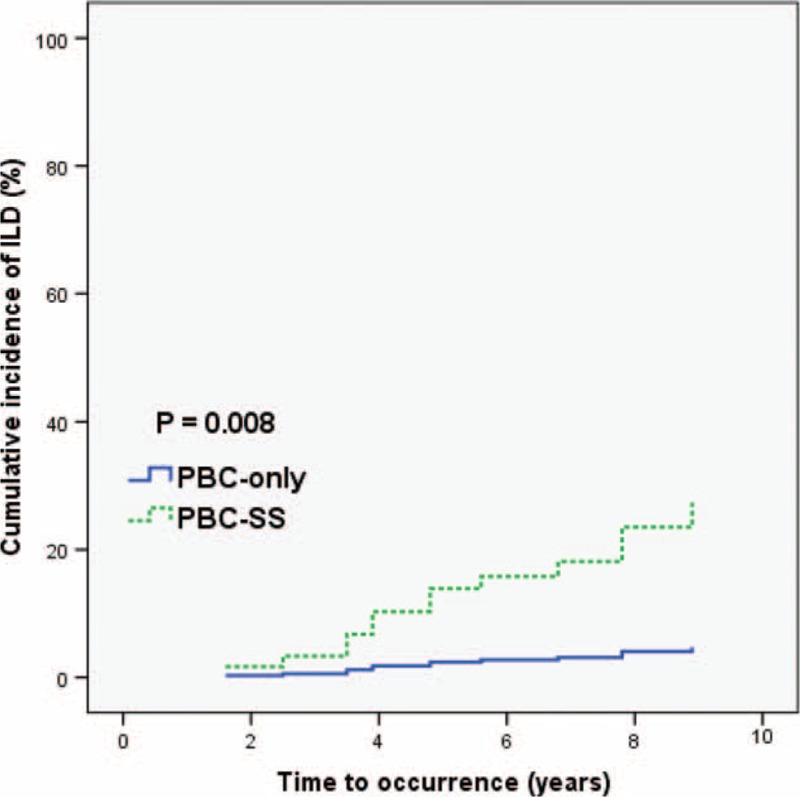
The cumulative incidence of ILD curves based on Kaplan–Meier method was significantly higher in primary biliary cirrhosis-Sjögren syndrome patients than in primary biliary cirrhosis-only patients. *P* = 0.008. PBC: primary biliary cirrhosis. PBC-SS: primary biliary cirrhosis with concomitant Sjögren syndrome. ILD: interstitial lung disease.

**FIGURE 3:**
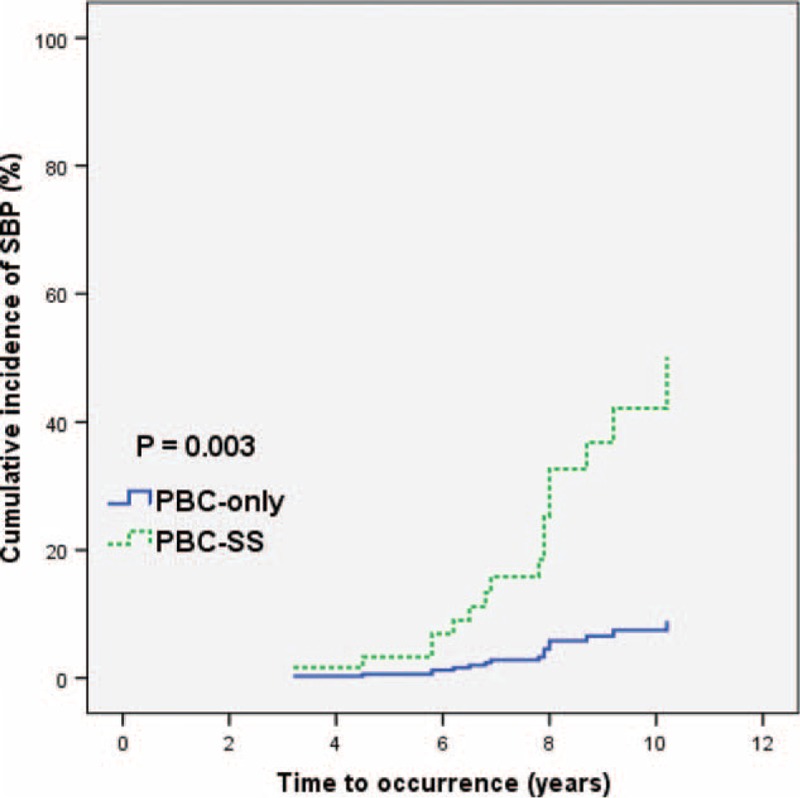
The cumulative incidence of spontaneous bacterial peritonitis curves based on Kaplan–Meier method was significantly higher in primary biliary cirrhosis-Sjögren syndrome patients than in primary biliary cirrhosis-only patients. *P* = 0.003. PBC: primary biliary cirrhosis. PBC-SS: primary biliary cirrhosis with concomitant Sjögren syndrome. SBP: spontaneous bacterial peritonitis.

**FIGURE 4:**
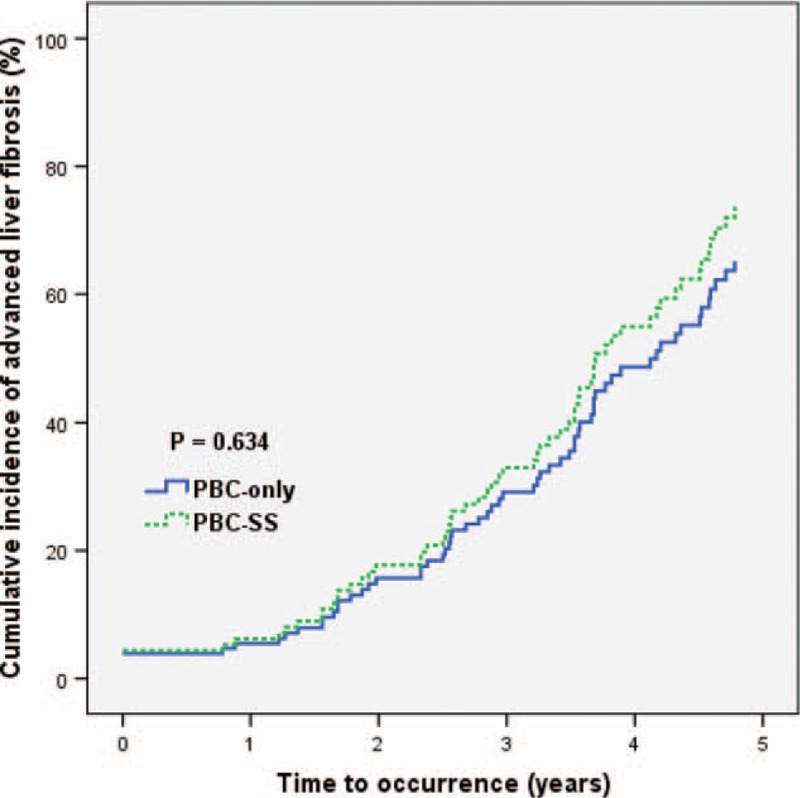
The cumulative incidence of advanced liver fibrosis curves based on Kaplan-Meier method was no significant difference between primary biliary cirrhosis-Sjögren syndrome and primary biliary cirrhosis-only patients. *P* = 0.634. Advanced liver fibrosis was defined as aspartate aminotransferase-to-platelet ratio index value >1.5. PBC: primary biliary cirrhosis. PBC-SS: primary biliary cirrhosis with concomitant Sjögren syndrome. APRI: aspartate aminotransferase-to-platelet ratio index.

After excluding patients with SLE and RA, the remaining 125 patients were divided into 2 groups according to the coexistence of SS. The analysis of 77 PBC-only patients and 48 PBC-SS patients is shown in Table 3. There were no significant differences in age, sex, and levels of albumin, international normalized ratio, creatinine, AST, alanine aminotransferase, γ-GT, ALP, TB, platelet count, TSH, free T4, APRI value, total cholesterol, triglyceride, low-density lipoprotein-cholesterol, and high-density lipoprotein-cholesterol between these 2 groups. The difference of outcomes between these 2 groups during the observation period is presented in Table 4. Death from all causes (*P* = 0.031), the risk of ILD (*P* = 0.005), and the incidence of SBP (*P* = 0.002) were all significantly higher in PBC-SS patients than in PBC-only patients. There were no significant differences in development of HCC, ESRD, variceal bleeding, and hypothyroidism. The overall survival curves between these 2 groups were statistically different (*P* = 0.033; Figure 1). All causes of death in PBC-only and PBC-SS patients were summarized in Table 5. In the PBC-only group, the causes of death included HCC (1 patient), myocardial infarction (1 patient), stroke (1 patient), and pneumonia secondary to ILD (1 patient). In the PBC-SS group, the causes of death included HCC (1 patient), pancreatic cancer (1 patient), hepatic failure (1 patient), ischemic bowel disease (1 patient), myocardial infarction (2 patients), and pneumonia secondary to ILD (3 patients). The cumulative incidence of ILD curves was significantly higher in the PBC-SS group than in the PBC-only group (*P* = 0.008; Figure 2), as was the cumulative incidence of SBP (*P* = 0.003; Figure 3). The 5-year cumulative incidence of advanced liver fibrosis was no significant difference between PBC-SS and PBC-only patients (*P* = 0.634; Figure 4).

**TABLE 3:**
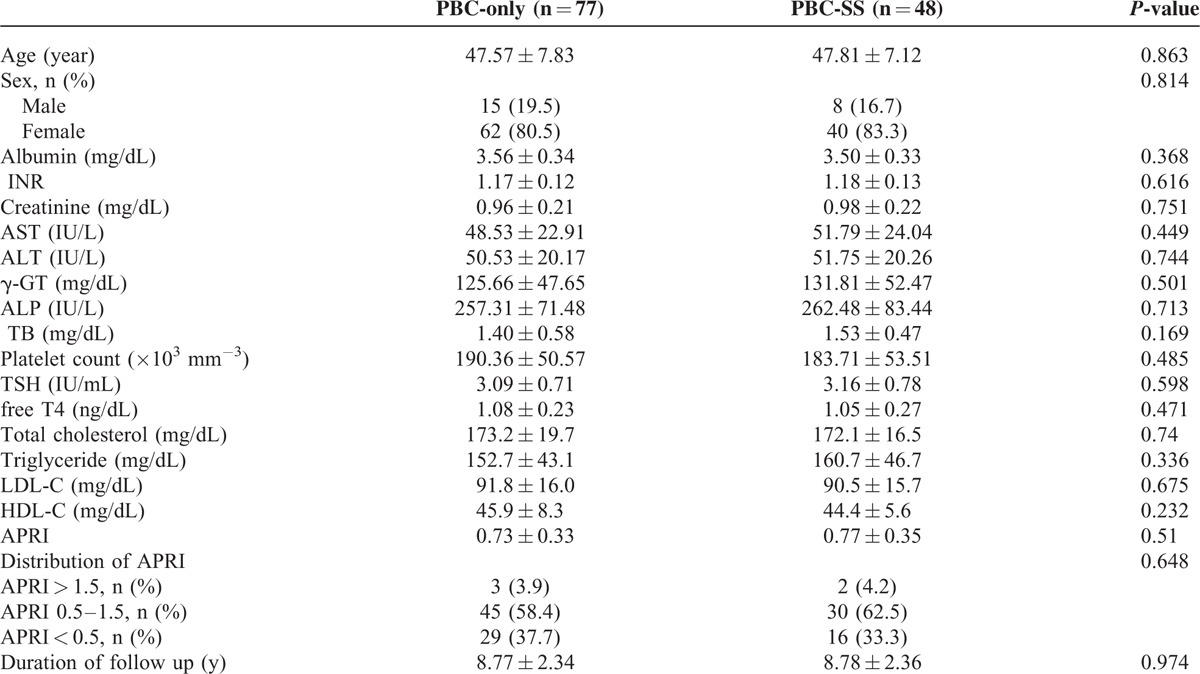
Clinical Characteristics of Primary Biliary Cirrhosis-only Patients and Primary Biliary Cirrhosis With Concomitant Sjögren Syndrome Patients

**TABLE 4:**
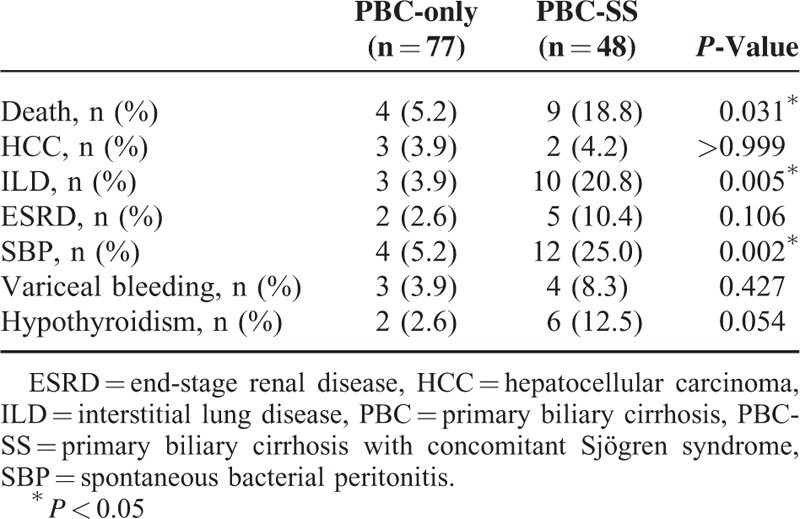
Comparison of Long-term Outcomes Between Primary Biliary Cirrhosis-only and Primary Biliary Cirrhosis With Concomitant Sjögren Syndrome Patients

**TABLE 5:**
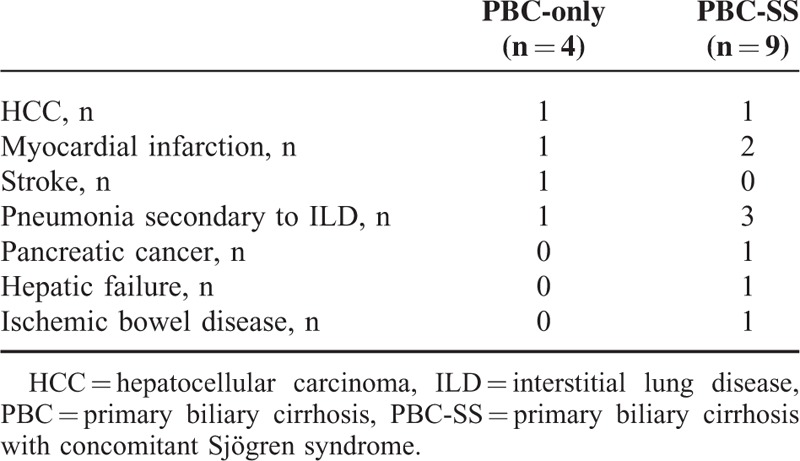
Causes of Death in Primary Biliary Cirrhosis-only and Primary Biliary Cirrhosis With Concomitant Sjögren Syndrome Patients

## DISCUSSION

Our study retrospectively evaluated the clinical characteristics and long-term outcomes of all patients with PBC. In subgroup analysis, the clinical characteristics and long-term outcomes between PBC-only patients and PBC-SS patients were compared. In our study, the incidence of SS in PBC is 35.3%, which is compatible with previous studies.^3,22^ The incidence of hypothyroidism tended to be higher in PBC-SS patients than in PBC-only patients. The incidence of SBP and ILD, and mortality rate in PBC-SS patients were significantly higher than in PBC-only patients. Our data suggested aggressive surveillance of thyroid and pulmonary functions in PBC-SS patients.

The high incidence of autoimmune thyroid disease (ATD) has been documented in SS patients. The most common presentation of ATD is subclinical hypothyroidism.^23^ In SS patients with concomitant ATD, the histologic findings from the thyroid gland are similar to those from the lacrimal and salivary glands, which demonstrate focal or diffuse T lymphocytic infiltration. The prevalence of thyroid disorders in SS patients is 14% to 45%.^5,24–26^ The incidence of ATD in PBC patients remains unclear. In our study, the incidence of hypothyroidism was nonsignificantly higher in the PBC-SS group than in the PBC-only group (12.5% versus 2.6%, *P* = 0.054). Longer follow-up duration and larger series may be conducted to evaluate the significant difference of the incidence of hypothyroidism between the 2 groups.

Renal insufficiency among SS patients has been well documented. Interstitial nephritis-related renal tubular acidosis is the most common presentation of renal disorders in these patients. Interstitial nephritis in these patients occurs with an incidence of 12% to 48%, and presents histologically as activated lymphocyte infiltration or immune complex-mediated damage in the renal tubular epithelium, which results in renal insufficiency.^7,27–29^ The influence of PBC on renal function is not well documented. In our study, the rates of ESRD in PBC-only patients and PBC-SS patients were 2.6% and 10.4%, respectively (*P* = 0.106). The causes of renal insufficiency in our study, however, could not be confirmed because renal biopsies were not performed.

The incidence of ILD in SS patients is reported to range from 8% to 38%. Histologic findings from lung parenchyma in ILD patients demonstrate alveolar septum thickening by fibrosis and lymphocytic infiltration. The development of ILD has been associated with a poor prognosis.^30,31^ Primary biliary cirrhosis was also reported to be associated with the development of ILD.^32^ In 1 large series by Shen et al, 28 of 178 PBC patients developed ILD. Of the 28 patients with PBC and ILD, 16 (57.1%) had concomitant connective tissue diseases, including 11 with SS. Autoimmune diseases are highly correlated with the incidence of ILD in PBC patients.^15^ In our study, the incidence of ILD was significantly higher in PBC-SS patients (20.8%) than in PBC-only patients (3.9%) (*P* = 0.005).

Spontaneous bacterial peritonitis is caused by bacterial migration from the gastrointestinal tract into ascites and by a deficiency of the immune system.^33,34^ Risk factors of SBP include advanced liver cirrhosis, low ascitic fluid protein levels, high serum TB levels, a history of SBP, variceal bleeding, malnutrition, and prolonged use of proton pump inhibitors.^35–37^ In advanced liver cirrhosis, serum complement deficiency and impaired phagocyte function were shown to be the main mechanisms of SBP.^35,38–40^ In our study, patients with advanced liver cirrhosis at initial diagnosis of PBC were excluded. The incidence of SBP significantly, however, increased in PBC-SS patients (*P* = 0.002) during the observation period. Although SS affects the complement system, the influence of the immune system in PBC-SS patients remains unclear.^4^ Our study demonstrated the high risk of developing SBP in PBC patients concomitant with SS, which indicated an aggravation of immune deficiency in PBC-SS patients. Further basic studies are needed to prove the possible mechanisms.

In our study, the overall survival rate was significantly higher in the PBC-only group than in the PBC-SS group, and 4 and 9 patients, respectively, died during the observation period. A total of 4 patients died because of ILD, including 1 PBC-only patient and 3 PBC-SS patients. Interstitial lung disease seems to be a more common cause of death in the PBC-SS group than in the PBC-only group, but the incidence is low. Longer observation times and larger studies are needed to evaluate the relationship between ILD and death in PBC-SS patients. Our study also indicates a poor prognosis in PBC-SS patients.

This study has several limitations. First, because this study is retrospective and not a randomized control trial, it may have unexpected bias. Second, thyroid aspiration biopsy samples, antithyroglobulin antibodies, and thyroid peroxidase antibodies were not collected in our study. Therefore, causes of hypothyroidism, such as autoimmune thyroiditis or subacute thyroiditis, could not be elucidated. Third, renal biopsy was not performed, and the pathophysiology of renal insufficiency could not be identified. Fourth, the observation time was short and sample size was small; longer follow-up studies with larger sample sizes would allow for observation of more significant differences, such as in the incidence of hypothyroidism, between the PBC-SS and PBC-only groups.

In the future, long-term prospective studies should be conducted to evaluate the clinical characteristics and differences in outcomes between the PBC-SS and PBC-only patients.

## CONCLUSIONS

The incidence of SS in PBC patients is high. During long-term observation, the risks of ILD and SBP are higher in PBC-SS patients than in PBC-only patients. The overall survival is lower in PBC-SS patients than in PBC-only patients. Our study indicates the poor prognosis of SS in PBC patients. Aggressive surveillance of thyroid and pulmonary functions is necessary for PBC patients with concomitant SS.
